# Complexity of VTA DA neural activities in response to PFC transection in nicotine treated rats

**DOI:** 10.1186/1743-0003-8-13

**Published:** 2011-02-27

**Authors:** Ting Y Chen, Die Zhang, Andrei Dragomir, Yasemin M Akay, Metin Akay

**Affiliations:** 1 Department of Biomedical Engineering, Cullen College of Engineering, University of Houston, Houston, TX 77204, USA

## Abstract

**Background:**

The dopaminergic (DA) neurons in the ventral tegmental area (VTA) are widely implicated in the addiction and natural reward circuitry of the brain. These neurons project to several areas of the brain, including prefrontal cortex (PFC), nucleus accubens (NAc) and amygdala. The functional coupling between PFC and VTA has been demonstrated, but little is known about how PFC mediates nicotinic modulation in VTA DA neurons. The objectives of this study were to investigate the effect of acute nicotine exposure on the VTA DA neuronal firing and to understand how the disruption of communication from PFC affects the firing patterns of VTA DA neurons.

**Methods:**

Extracellular single-unit recordings were performed on Sprague-Dawley rats and nicotine was administered after stable recording was established as baseline. In order to test how input from PFC affects the VTA DA neuronal firing, bilateral transections were made immediate caudal to PFC to mechanically delete the interaction between VTA and PFC.

**Results:**

The complexity of the recorded neural firing was subsequently assessed using a method based on the Lempel-Ziv estimator. The results were compared with those obtained when computing the entropy of neural firing. Exposure to nicotine triggered a significant increase in VTA DA neurons firing complexity when communication between PFC and VTA was present, while transection obliterated the effect of nicotine. Similar results were obtained when entropy values were estimated.

**Conclusions:**

Our findings suggest that PFC plays a vital role in mediating VTA activity. We speculate that increased firing complexity with acute nicotine administration in PFC intact subjects is due to the close functional coupling between PFC and VTA. This hypothesis is supported by the fact that deletion of PFC results in minor alterations of VTA DA neural firing when nicotine is acutely administered.

## Background

The mesocorticolimbic dopamine system, consisting of the ventral tegmental area (VTA), prefrontal cortex (PFC) and nucleus accumbens (NAc), is a critical substrate for the neural adaptations that underlie addiction [1]. The dopamine (DA) neurons in VTA and their projection areas, including PFC, NAc, and amygdala, are thought to be very important in the reward driven behavior induced process by the drugs of addiction [1-5]. Nicotine is a biologically active substance that promotes tobacco use and has caused the global population health and economical problems. Unfortunately, nicotine dependence creates problems for smokers to quit. The mesocorticolimbic dopamine pathways have been shown to be stimulated by nicotine. The stimulation originates from VTA and resulting in DA secretion within the NAc and PFC is essential for the reinforcing effects of nicotine [6]. Moreover, other neurotransmitter pathways like glutamatergic neurons projecting from PFC to VTA are also involved in the motivational effects of nicotine [7,8]. The important role played by glutamatergic pathways in excitation of mesocorticolimbic dopaminergic neurons by nicotine has been demonstrated by many previous studies [9].

The firing activities of VTA DA neurons and addictive behavior of the animals are believed to be controlled by the glutamatergic synaptic inputs from PFC [10-14]. The PFC is a key structure for executive functions of the brain [15,16], and has been shown to regulate the firing pattern of VTA DA neurons. Therefore, the burst firing in VTA DA neurons increases with PFC stimulation and the opposite effect is shown with PFC inactivation [17-21]. The strengthening of input from PFC to VTA plays an important role in the behavioral sensitization development, a well-known model for addiction [22-24]. Evidence has shown the functional input loss from PFC and/or NAc may reduce the effects of these drugs on the addiction process [13,25-27]. Studies have demonstrated that under *in vivo *conditions, the VTA DA neurons produce single spikes and/or burst firing. Additionally, they are capable of firing in a slow oscillatory (SO) pattern. The SO generation needs inputs from other brain area (i.e. PFC) [28,29].

Previous studies show that systemic nicotine injection can increase the firing rate and percentage of bursting firing of VTA DA neurons [30-33]. However, the PFC transection only excited 28% of the VTA DA neurons which could be stimulated only by systemic nicotine activation, but not by the PFC [32,33]. Also, we have known that VTA DA neurons' bursting firing mode needs excitatory inputs. Therefore, we hypothesize that systemic exposure to nicotine significantly affects the complexity of firing of the VTA DA neuron and this alteration should be based on the intact input from other brain areas. Since PFC is the main source of excitatory inputs to the VTA, the effect of nicotine on the complexity of VTA DA neuronal firing will be reduced, when the pathway between PFC and VTA is disconnected. To test this hypothesis, we recorded VTA DA neurons firing and analyzed the data using the advanced nonlinear dynamical analysis method based on the Lempel-Ziv (LZ) estimator.

Traditional analysis methods of neuronal firing activity consist only in measuring spike amplitude and/or extracting spike frequency information in order to characterize the changes produced in the VTA or other brain areas by different physiological factors or pharmacological treatments [34,35]. However, the use of such methods often renders comparisons within subject groups not possible. The amplitude characteristics or frequency of rhythms may differ from subject to subject. Additionally, they may not offer any insight on the firing patterns generated by the neural activity. Therefore, more robust and meaningful analysis methods need to be used for the dynamical analysis of neural recordings. The dynamical analysis is especially relevant in the context of VTA DA neurons, which are part of neural networks that receive inputs from several other brain areas. Therefore, in this study, we have analyzed the dynamics (complexity) of nicotine-induced neuronal firing pattern in the VTA DA neurons in both PFC intact and transected Sprague Dawley (SD) rats using the Lempel-Ziv (LZ) method. We also estimated the entropy values of the recorded firing activity and compared the results obtained from LZ analysis and entropy [36].

## Methods

### Electrophysiological recordings

All experimental protocols and surgeries were approved by The Institutional Animal Care and Use Committee of Arizona State University. We used male Sprague-Dawley (SD) rats from Charles River Laboratories (Wilmington, MA) weighting between 250 and 300 grams. All animals were anesthetized with chloral hydrate (400 mg/kg, intraperitoneal (i.p.) injected) and mounted with stereotaxic apparatus (Narishige, Japan) for extracellular single-unit recording. The extracellular recording pipette was filled with 2 M NaCl (Sigma) and 0.5% Chicago sky blue (Sigma) solution and placed into the VTA through a small burr hole in the skull (2.7-3.3 mm anterior to the lambda and 0.5-0.9 mm lateral to the midline) by an electro-microdriver. DA neurons, usually at 6.5-8.5 mm below the cortical surface, were identified according to the well established electrophysiological criteria [37-41]. After stable recording was established for a minimum of five minutes as baseline, (-) nicotine hydrogen tartrate salt (Sigma Chemical Co., St. Louis, MO) at a smoking-relevant concentration (0.5 mg/kg, i.v. via tail vein) was administered and recordings were continued for at least 15 minutes. Mereu et al [42] studied the influence of various doses of nicotine on Dopamine (DA) neurons in rats either general or local anesthesia. Their results showed the optimal dose of nicotine (0.5 mg/kg) that produced a significant increase in the firing rate of DA neurons. Stolerman et al performed similar studies [43] that confirmed that 0.5 mg/kg was an optimal and effective dose to study the influence of nicotine in the neural firings of DA neurons. Many others [31,44,45] also used 0.5 mg/kg dose of nicotine to study the behavior of DA neurons. Therefore, these studies encouraged us to focus on the single, optimal dose to investigate the influence of nicotine on the dynamics of neural firings of DA neurons. The body temperature was maintained at 36 to 38°C. The recording sites were marked by ejection of Chicago sky blue and examined using standard histology methods at the end of experiments [32,33,40].

### PFC transection

To study the interaction of PFC inputs to the VTA DA neurons, bilateral transections were made immediate caudal to the PFC to disrupt the communication between PFC and VTA DA neuron. A slit was drilled in the skull 2.0 mm anterior to bregma. Without damaging the main artery, a sharp blade was lowered to the base of skull, to completely interrupt the connections between the PFC and the rest of the brain. All surgical procedures were done under anesthetized condition [32,33,40].

### Data acquisition and analysis

The firing activities of VTA DA neurons were recorded from five SD rats for both PFC intact and PFC transected rats. Data was acquired and recorded on the same data acquisition system (Powerlab, ADInstruments). We quantified the neural dynamics using the LZ complexity estimator as detailed below. Two-minute segment of data before the injection of nicotine was analyzed with LZ complexity method. After firing rate of DA neuron has reached stable condition in response to nicotine, two minutes of data with the effect of nicotine was analyzed with LZ complexity to understand the dynamics (complexity) of neural firing in response to nicotine exposure to VTA DA neurons with and without input from PFC. All values are expressed as mean ± SEM. Statistical significance was assessed using paired two-tailed Student's *t*- tests.

### Lempel-Ziv Complexity

The firing activity recorded from VTA DA neurons arises from complex feedback networks and nonlinear interconnections, which are characteristic for such neural systems. Therefore, we used the LZ estimator as a measure of complexity (regularity) of the firing activities recorded from VTA DA neurons [46-49]. LZ complexity is closely related to information-theoretical methods such as entropy [48] and is able to cope with discrete-time symbolic sequences. It quantifies the rate of new pattern generation along given sequences of symbols. The symbolic representations of time series are particularly favored when low-amplitude noise hampers the data [49].

Therefore, we transformed the neural signals into a finite sequence in the symbolic space. Each sample in the time domain was assigned a symbol, and the total number of unique symbols formed the alphabet of the sequence. Since the data was composed of a series of action potentials that form the response of the neurons to the input, we used a binary alphabet. The time axis was divided into discrete bins. The action potentials were detected using an amplitude threshold, and each time the threshold was crossed, we placed a "1" in the respective bin of the symbolic representation of our signals. All bins with values below the threshold were assigned a "0" [49].

Formally, our signal *x*(*n*) was converted into a binary sequence *S *= *s*(*1*), *s*(*2*), ..., *s*(*n*), where

(1) $$s(i)=\left\{ \begin{array}{l} 0\begin{array}{cc} , & \end{array}\begin{array}{cc} if & \end{array}x(i)<T\\ 1\begin{array}{cc} , & \end{array}otherwise, \end{array}\right.$$

where *T *is the threshold and can be chosen as 2SD(*x*(*n*)), where SD(*x*(*n*)) represents the standard deviation of the original signal *x*(*n*) [49].

For computing the LZ complexity, the sequence *S *is parsed from left to right, and a complexity counter *c*(*n*) is increased each time a new subsequence (distinct word) is encountered. The algorithm followed is:

• Let *S*(*i, j*) denote a substring of S that starts at position *i *and ends at position *j*, where *i < j*. *S*(*i, j*) = *s_i _s_i+1 _*... *s_j _*and when *i > j*, *S*(*i, j*) = {}. The vocabulary of the sequence *S*, *V*(*S*), is the set of all unique substrings (words) *S*(*i, j*) of *S*.

• The parsing procedure starts by comparing a substring *S*(*i, j*) to the vocabulary that is comprised of all substrings of *S *up to *j - 1*, that is *V*(*S*(*1, j - 1*)). If *S*(*i, j*) is present in *V*(*S*(*1, j - 1*)) then update *S*(*i, j*) and *V*(*S*(*1, j - 1*)) to *S*(*i, j + 1*) *V*(*S*(*1, j*)), respectively, and repeat the previous check. If the substring is not present, place a dot after *S*(*j*) to indicate the end of a new component, update *S*(*i, j*) and *V*(*S*(*1, j - 1*)) to *S*(*j + 1, j + 1*) and *V*(*S*(*1, j*)), respectively, and the process continues. The whole parsing operation begins at *S*(*1,1*) and continues until *j = n*, the total length of the binary sequence [47].

For example, the sequence *S *= 1011110100010 is parsed as 1 .0 . 11 . 110 . 100 . 010. Therefore, the vocabulary of *S *is six. Similarly, a sequence *S *= 0001101001000101 would be parsed as 0 . 001 . 10 . 100 . 1000 . 101, and hence yields a vocabulary sized six [46].

LZ complexity is defined as the total number of words in the decomposition, *c(n)*. The normalized LZ complexity is defined as

(2) $$C_{LZ}=\frac{c(n)}{n/\log_{2}n}.$$

More details on the LZ method and its implementation are given elsewhere [46-50].

### Entropy

In addition to the LZ estimator, we also analyzed the same data set using the approximated entropy (complexity) since it has been widely used for the analysis of biomedical signals. The entropy estimates can be computed as follows [36]:

(3) $$H=-\sum_{n}p(n)\cdot \log_{2}p(n)$$

Where *p*(*n*) is the probability of observing *n *spikes in the time window. The time resolution was 10 ms and entropy was computed on segments of 20 s length.

## Results

To evaluate the firing pattern changes of VTA DA neurons to systemic nicotine exposure, the extracellular single-unit recordings were performed in DA neurons in anesthetized rats as described in methods section. Two minutes of data was divided in 20-second windows for analysis purposes. LZ complexity was estimated for each 20-second window and the values were averaged. The same procedure was applied for segments before and after nicotine exposure. The data analyzed for nicotine effect was taken after firing rate of DA neuron has reached stable condition in response to nicotine administration.

**Figure 1:**
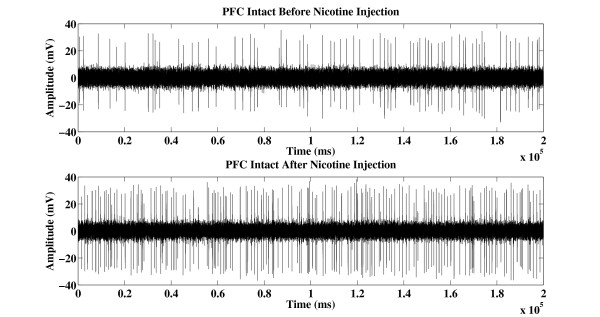
**Example action potential recorded from PFC intact VTA DA neuron of SD rat before and after nicotine injection**.

**Figure 2:**
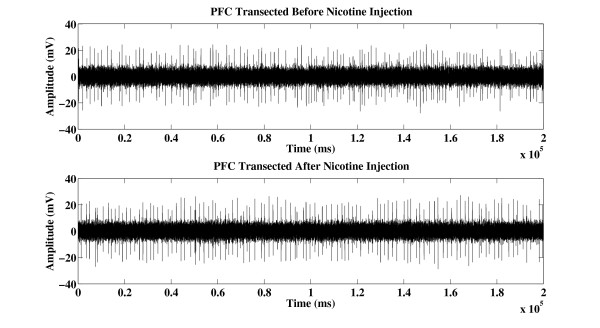
**Example action potential recorded from PFC transected VTA DA neuron of SD rat before and after nicotine injection**.

**Figure 3:**
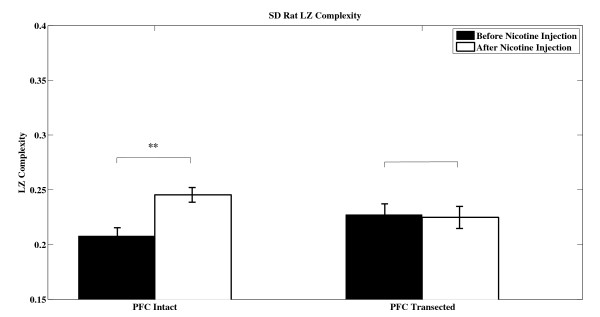
The mean LZ complexity values ± SEM of five intact SD rats and five transected SD rats before and after nicotine exposure (** indicates *p *< 0.01, paired two-tailed Student's *t*-test).

Figure 1 shows an example of 20-second segment action potential recorded from PFC intact VTA DA neuron before and after nicotine injection. Figure 2 shows an example of 20-second segment action potential recorded from PFC transected VTA DA neuron before and after nicotine injection. Both firing rate and firing pattern look similar when observed with naked eye. The left panel of Figure 3 shows the averaged LZ complexity values from five PFC intact SD rats before and after nicotine administration. The right panel of Figure 3 shows the averaged LZ complexity values from five PFC transected SD rats before and after nicotine administration.

The LZ complexity values were 0.2079 ± 0.0075 before nicotine administration and were 0.2454 ± 0.0067 after nicotine administration for SD rats with PFC intact. As shown in Figure 3, there is significant increase in the complexity values in DA neurons after nicotine exposure (*p *< 0.01) for PFC intact rats. Figure 3 indicates that nicotine plays an important role in affecting the firing of DA neurons in VTA. Considering that the excitatory input to VTA DA neurons is mainly originated from the PFC, the above results suggests a possibility that systemic nicotine-induced changes of VTA neuron firing might be mediated through an alteration in PFC neural function. To test this hypothesis, we interrupted the PFC and VTA interaction by acute PFC transection. The transection was done mechanically immediate caudal to the PFC by acute transecting both sides of PFC as described in methods. The LZ complexity values were 0.2273 ± 0.0099 before nicotine administration and were 0.2248 ± 0.0101 after nicotine administration for SD rats with PFC transected. As shown in Figure 3, there is no significant difference (*p *= 0.8085).

**Figure 4:**
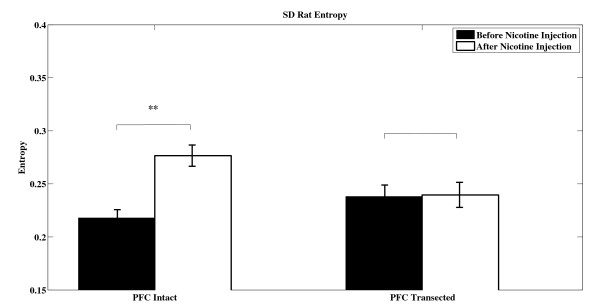
The mean entropy values ± SEM of five intact SD rats and five transected SD rats before and after nicotine exposure (** indicates *p *< 0.01, paired two-tailed Student's *t*-test).

In addition to LZ complexity analysis method, we also calculated entropy estimates of the same neural recordings for comparison purposes. The entropy values were 0.2179 ± 0.0078 before nicotine administration and were 0.2766 ± 0.0100 after nicotine administration for SD rats with PFC intact. As shown in Figure 4, there is a significant increase in the entropy values in DA neurons after nicotine exposure (*p *< 0.01) for PFC intact rats. The entropy values were 0.2382 ± 0.0107 before nicotine administration and were 0.2396 ± 0.0118 after nicotine administration for SD rats with PFC transected. As shown in Figure 4, there is no significant difference (*p *= 0.9319).

## Discussion and conclusion

In this study, we used nonlinear dynamical analysis methods based on the LZ method and the approximated entropy to analyze VTA DA neuronal firing activity induced by systemic administration of nicotine on PFC intact and transected rats. The analyses allow us to quantitatively distinguish the firing patterns dynamics of VTA DA action potentials. These patterns may reflect different status of neuronal network synchronization. Nonlinear dynamical analysis of neural patterns demonstrated that nicotine only significantly affects PFC intact rats and this may be due to the close connection between PFC and VTA.

The neural activity recorded from VTA DA neurons arises from complex networks and non-linear interconnections, which are neural systems characteristics. The fact that the neural activity arises from such complex systems, as well as the symbolic-like features of the recorded data, make the use of the LZ complexity measure suitable in the context of the present work [51-68].

Provided by its robustness over other complexity/entropy measures, the LZ complexity has been applied extensively in biomedical signal analysis as a metric to estimate the complexity of discrete-time physiologic signal. For example, LZ has been used for recognition of structural regularities [54], for complexity characterization of DNA sequences [57-59], to develop new methods for discovering patterns in DNA sequences by applying it to genomic sequences of Plasmodium falciparum [59], and to estimate the entropy of neural discharges (spike trains) [48,60]. LZ complexity has also been used to study brain function [62], brain information transmission [63], EEG complexity in patients with Alzheimer's disease [64], epileptic seizures [65], ECG dynamics [66], and to evaluate the nature and dynamics of hippocampal neuronal oscillations [50,69,70].

In recent studies the performance of the LZ estimator was compared to other entropy measures for the analysis of the biomedical signals [49,71]. Although LZ complexity was shown to be related to entropy [48,68], it proved to be less sensitive to the length of data [71]. Its better performance in terms of sensitivity to signal bandwidth changes was also reported, when compared to Shannon entropy [71].

All these previous studies encouraged us to use this nonlinear dynamical analysis method, based on the LZ complexity method, to gain insights into the VTA DA neuronal activity induced by systemic administration of nicotine to both PFC intact and transected subjects. The results obtained when using the LZ estimator were confirmed by those obtained when the entropy of the neuronal firing was estimated. Therefore, our results confirm our hypothesis that nicotine significantly affects the firing of VTA DA neurons and that this effect is based on the intact input from PFC.

The increase of the excitatory drive onto the DA neurons is activated by presynaptic terminals of glutamatergic afferents induced by nicotine [31,72,73]. This potentiated glutamatergic drive causes DA neurons to fire more in a burst or phasic mode [30,31], since the firing rate and pattern of VTA DA neurons change with nicotine exposure. We speculate the increased complexity in PFC intact subject is due to a close functional coupling between PFC and VTA and the increased neural activity in VTA DA neurons. Our analysis demonstrated that the complexity/entropy values of neural activity after nicotine exposure were significantly increased when the connection between PFC and VTA is intact. On the other hand, the complexity/entropy values have no significant change when the input from PFC to VTA is disconnected. The reason for the increased complexity and entropy is the increased neural activity resulted from nicotine exposure.

The PFC and VTA have close functional coupling. Stimulation of PFC increases burst firing in VTA DA neurons, while deletion of PFC induces the opposite effect [17-19,21,74]. Gao et al [40] reported that under non-stimulation conditions, the activity of VTA DA neurons co-varied with PFC neuronal activity, suggesting a close functional coupling between PFC and VTA [40]. Evidence indicates a key control of VTA neuronal function by PFC [38].

Our analysis indicates that the LZ estimators and entropy are useful tools for the characterization of the dynamical changes in VTA DA neuronal activity. As demonstrated in our analysis, such changes could be quantitatively represented as an impairment of neuronal firing during nicotine exposure and PFC transection.

## Competing interests

The authors declare that they have no competing interests.

## Authors' contributions

TC performed experiments and the data analysis and helped to write the manuscript, DZ helped with the experiments and helped to write the manuscript, AD contributed to the data analysis and helped to write the manuscript, YMA helped with the experiments and helped to write the paper. MA oversaw the data collection, the data analysis, and helped to write the manuscript. All authors read and approved the final manuscript.
